# The WorkingWell Mobile Phone App for Individuals With Serious Mental Illnesses: Proof-of-Concept, Mixed-Methods Feasibility Study

**DOI:** 10.2196/11383

**Published:** 2018-10-25

**Authors:** Joanne Nicholson, Spenser M Wright, Alyssa M Carlisle, Mary Ann Sweeney, Gregory J McHugo

**Affiliations:** 1 Institute for Behavioral Health The Heller School for Social Policy and Management Brandeis University Waltham, MA United States; 2 Department of Psychiatry Dartmouth-Hitchcock Medical Center Lebanon, NH United States; 3 Department of Psychiatry The Geisel School of Medicine at Dartmouth Lebanon, NH United States

**Keywords:** mHealth, mobile apps, mental disorders, employment

## Abstract

**Background:**

The disparities in employment for individuals with serious mental illnesses have been well documented, as have the benefits of work. Mobile technology can provide accessible in-the-moment support for these individuals. The WorkingWell mobile app was developed to meet the need for accessible follow-along supports for individuals with serious mental illnesses in the workplace.

**Objective:**

We explore the usability, usage, usefulness, and overall feasibility of the WorkingWell mobile app with individuals with serious mental illnesses who are actively employed and receiving community-based services.

**Methods:**

In this proof-of-concept, mixed-methods, 2-month feasibility study (N=40), employed individuals with serious mental illnesses were recruited in mental health agencies. Participants completed surveys regarding background characteristics and cellphone use at enrollment and responded to interview items regarding app usability, usage, and usefulness in technical assistance calls at 1, 2, 4, and 6 weeks of participation and in the exit interview at 8 weeks. Data on the frequency of app usage were downloaded on a daily basis. A version of the System Usability Scale (SUS) was administered in the exit interview. Overall feasibility was determined by the percent of users completing the study, responses to an interview item regarding continued use, and findings on usability, usage, and usefulness. General impressions were obtained from users regarding user support materials, technical assistance, and study procedures.

**Results:**

Most participants were male (60%, 24/40), aged 55 or younger (70%, 28/40), white (80%, 32/40), had less than a 4-year college education (78%, 31/40), were employed part-time (98%, 39/40), had been working more than 6 months (60%, 24/40), and indicated a diagnosis of bipolar, schizoaffective, or depressive disorder (84%, 16/25). The majority of participants owned cellphones (95%, 38/40) and used them multiple times per day (83%, 33/40). Their average rating on SUS usability items was 3.93 (SD 0.77, range 1.57-5.00), reflecting positive responses. In general, participants indicated WorkingWell was “very easy,” “straightforward,” “simple,” and “user friendly.” Usability challenges were related to personal issues (eg, memory) or to difficulties with the phone or app. Data on app usage varied considerably. The most frequent navigations were to the home screen, followed by Rate My Day and My Progress, and then by Manage the Moment and Remind Me. The app was described as useful by most participants; 86% (30/35) agreed the app would help them manage better on the job. Of the 40 original participants, 35 (87%) completed the study.

**Conclusions:**

The WorkingWell app is a feasible approach to providing accessible, as-needed employment support for individuals with serious mental illnesses. The app would benefit from modifications to address recommendations from feasibility testing. Controlled research with larger samples, more diverse in individual characteristics and workplace settings, is essential to demonstrating the effectiveness of the app.

## Introduction

### Supporting Employment

The disparities in employment for individuals disabled by mental illnesses such as schizophrenia, bipolar disorder, and major depressive disorder have been well documented [1-4], as have the benefits of work for these individuals [5-9]. Employment provides daily activity and routine, and opportunities for building social supports, with positive impact on self-esteem and independence, social integration, and community participation. Supported employment service delivery models, such as Individual Placement and Support (IPS), have demonstrated robust success in promoting competitive employment among individuals with psychiatric disabilities [10]. IPS promotes consumer choice and shared decision making regarding employment plans, collaborative involvement of consumers with the treatment team to identify and implement strategies to promote success and competence in finding competitive work, and ongoing support to help maintain a positive employment course. The effectiveness of IPS for individuals with severe psychiatric disabilities has been established in 17 randomized controlled trials [11-13]. Approximately two-thirds of clients enrolled in IPS achieve the goal of a competitive job compared to fewer than one-quarter of clients who receive other forms of vocational services [14].

Although supported employment services have demonstrated effectiveness in helping individuals achieve the goal of competitive employment [6,15], sustaining employment presents additional challenges [8,16]. Symptom severity and limitations in neurocognitive capacities, interpersonal skills, motivation to work, and self-efficacy undermine job tenure. In-person supported employment services are not routinely provided on the job, creating a gap in support for individuals with mental illness who are actively working.

The use and benefits of mobile technology in providing accessible, in-the-moment support for individuals with mental illnesses have been demonstrated [17-21]. Individuals with mental illnesses rely on Web- and technology-based health information and tracking tools, just as do individuals who are well [20-26], particularly if tools are appropriately designed and adequate training is provided [27,28]. The groundwork has been laid for technology-based tools to have a positive impact for individuals coping with challenges in the workplace [29,30]. In focus groups of supported employment service recipients conducted in the discovery phase of this study, individuals living with serious mental illnesses reported work challenges related to job characteristics, tasks, and expectations; interpersonal and social situations; illness- and treatment-related issues; lifestyle/wellness and conditions apart from work; and sustaining motivation [30]. The majority of participants owned mobile phones and were comfortable using technology.

### The WorkingWell App

The WorkingWell mobile app was developed in response to the need for accessible follow-along supports for individuals with serious mental illness who are actively employed [31]. The app was developed through the collaborative efforts of researchers, providers, individuals with serious mental illnesses, an Expert Advisory Panel (including supported employment services trainers and providers), and experienced app designers. It was informed by user experience design. Iterative cycles of usability testing were conducted individually, side-by-side, and in focus groups as content, information architecture, and navigation were developed. The principles underpinning the app were drawn from evidence-based supported employment [32]. Motivational and behavior change theories and strategies were actively embedded in WorkingWell features and functions through content development as well as in the design of interactions and feedback.

Users begin their interactions with WorkingWell by setting up to three work-related goals each week, selected from a prepopulated list or by adding their own. They are provided a motivational quote and image and are reminded of their goals each day they access the app. Users are encouraged to choose new goals each week. Once they have chosen or reviewed their goals, users navigate to the home page, where they find the four main app components: Manage the Moment, Remind Me, Rate My Day, and My Progress. In Manage the Moment, coping skills and tips for dealing with challenges are provided, along with ideas for how to implement selected coping strategies. Remind Me provides tools that are built into the app for setting text message reminders, creating to-do lists, and making notes. In Rate My Day, users rate their effort in accomplishing their goals, from 1 to 5 stars, along with rating their success in other areas such as dealing with stress and finishing tasks. My Progress provides feedback based on users’ ratings (eg, “Way to go! Things are going fantastic! What can you do to keep it up?”) and a detailed record of their entries for the past 4 weeks, so users can track their progress and explore patterns in their work day activities and evaluations.

### Research Questions

In this study, we explore the feasibility of use of the WorkingWell app by individuals with serious mental illnesses receiving community-based supported employment services and actively working. Research questions include:

1. Do study participants find WorkingWell easy to use (*usability*)?
2. Do study participants use the app, and which components are used most frequently (*usage*)?
3. Do they find the WorkingWell app useful with regard to managing work demands and illness issues, and which specific app features or components are most useful (*usefulness*)?
4. Is the WorkingWell app a reasonable, practical tool capable of being used by individuals with serious mental illnesses in dealing with employment challenges (*feasibility*)?

Recommendations for improvements in the WorkingWell app are solicited from study participants. Findings will inform ongoing refinements to WorkingWell and suggest future implementation approaches and research targets for individuals with serious mental illnesses as well as individuals coping with other health challenges at work.

## Methods

### Study Design

In this proof-of-concept, mixed-methods feasibility study, we addressed questions related to the usability, usage, usefulness, and overall feasibility of the WorkingWell mobile support tool (“app”) for working adults with serious mental illness. A complete description of the study protocol, methods, and procedures was previously published [31]. This previous publication included images of the WorkingWell app along with the WorkingWell User Guide, published as supplementary material [31]. The WorkingWell team implementing the study included the principal investigator (a doctoral-level clinical and research psychologist) and two research staff members with undergraduate degrees in social sciences and previous experience in research, trained by the principal investigator in procedures and methods relevant to the study. The researchers did not have preestablished relationships with nor provided services to study participants.

### Recruitment

#### Participant Eligibility and Screening

A convenience sample of adults with serious mental illnesses was enrolled from six community mental health agencies in Massachusetts, Vermont, and Maryland. Criteria for study enrollment included that participants had to be (1) 18 years of age or older; (2) receiving supported employment services (and, by definition of service eligibility and disability, living with serious mental illness); (3) working an average of 10 or more hours per week; (4) employed in a position that was not, by definition, seasonal or temporary; and (5) capable of reading and writing in English at a sixth-grade reading level or higher. Participants could have been employed for any length of time at the point of study enrollment, given our interest in the usefulness of the app at various times in the employment trajectory. Participants were not required to have a minimum level of familiarity with mobile phone or computer technology to enroll in the study, as the relationship between WorkingWell use and variation in experience with mobile phones was an issue to be explored. Participants received a stipend for completing the study orientation and enrollment interview (US $25), midway during the 8-week study period at the completion of the fourth technical assistance call (US $50), and at their exit interview from the study (US $75). Agency staff members, designated as liaisons, assisted in distributing information about the study and screening individuals volunteering to participate. Research staff reviewed participants’ eligibility criteria with agency liaisons prior to enrolling participants in the study.

#### Sample Size

Forty participants with serious mental illness were enrolled in the study. This was determined to be an adequate sample size given the study focus on feasibility. Forty participants allowed us to investigate the range of ways in which individuals experienced using the app.

### Procedures

#### Orientation Session and Enrollment Data Collection

Research staff traveled to agency sites to enroll participants and provide an in-person orientation to the study, mobile phone, and WorkingWell app. Orientations were conducted as individual or group sessions (up to eight participants) depending on the number of participants recruited at a particular site and participant availability. Orientation sessions varied in length due to differences in group size, lasting about 1.5 hours on average. Staff first described the study and obtained written informed consent to participate from attendees. Participants were assigned unique study identification numbers, and they completed the paper-and-pencil enrollment survey.

Participants were then provided with Android mobile phones with unlimited data plans to access WorkingWell and communicate with research staff. Phones were provided to ensure the app was implemented by all participants using a standard interface and operating system, to facilitate the staff’s ability to provide technical assistance, and to avoid creating a financial burden or barrier to study participation.

In the orientation session, research staff reviewed mobile phone and app navigation and functions using study phones. A Study Phone User Guide and WorkingWell User Guide [31] were provided to each participant. Participants were offered individualized hands-on technical assistance by researchers if required. Participants engaged in a discussion of appropriate mobile phone use in the workplace (eg, using WorkingWell during a lunch break or before or after work rather than while on the job if employment policies precluded phone use during work hours) to discourage phone or app use that would negatively impact their employment or safety.

#### Technical Assistance Calls

Research staff provided technical assistance to study participants on the telephone one day after the orientation session and during weeks 1, 2, 3, and 6 of study participation. The time and location of the calls were determined by participants (eg, after work hours or during a work break). At the start of each call, research staff confirmed with participants that it was a convenient time and that they were in a safe, comfortable environment (eg, not driving or distracted by environmental stimuli). Questions and prompts focused on challenges in using the mobile phone or app, general impressions of the app, how the app was used at work in the past few days or anticipated use the next time at work, confidence in using the app, and any additional support or information required. In technical assistance calls 2 through 5, additional prompts were added to obtain greater detail regarding app use, including the ease of use and usefulness of specific app features and components, and the ways in which WorkingWell was incorporated into the participant’s daily schedule. Responses to technical assistance call prompts were recorded verbatim by research staff using standardized forms developed for the study. If participants missed two consecutive technical assistance calls or were out of communication with research staff for more than 3 weeks, they were considered lost to follow-up.

Participants could also access research staff members as needed by telephone call or text message. Participant-initiated communications with researchers most often related to the scheduling of technical assistance or exit interview sessions, report of a problem with the app or the study phone, request for technical assistance for specific issues, or coordination of study incentive retrieval. These calls, while infrequent, were logged in detail as memos by research staff to provide complete data on any challenges faced by participants in phone or app use. For individuals who required additional help, in-person assistance was provided at the agency site by the research staff or agency liaison.

#### Exit Interviews

Exit interviews were completed with participants in person, in meeting rooms at agency sites at the end of the 8-week study period. Participants completed a poststudy paper-and-pencil survey. Additional open-ended interview items focused on user experience of the app and impressions of the research experience. Responses to exit interview questions were recorded in detail by research staff in a standardized format that included a section for additional observations and field notes. All exit interviews were completed individually with participants except in two instances. One participant confirmed that the agency liaison could be present, and another participant wished for her mother to attend the session. These invited individuals did not directly participate in the interview in any way.

### Measures

#### Participant Characteristics

Participants completed survey items regarding background and demographic characteristics at the time of study enrollment. These included questions about age, gender, race, education, employment, living situation, marital and family status, and mental health diagnosis. They completed a set of items regarding their access to, type, and frequency of cellphone use; six other items related to ease of phone use (eg, typing, sending a text message, accessing the internet, using an app, taking pictures, and using social networking sites) were rated on a 5-point scale from 1 (“can’t do at all”) to 5 (“really well”), except for ease of typing, which was rated on a scale from 1 (“not at all easy”) to 5 (“extremely easy”). These items were adapted from a prior study of mobile technologies and people with serious mental illness [17].

#### Usability

During the exit interview, participants completed the poststudy survey. Usability was assessed by an adapted version of the System Usability Scale (SUS) [33-35]. A subset of seven SUS items was determined to be most relevant to the study. Participants’ responses were rated on a 5-point Likert-type scale (1=“strongly disagree” to 5=“strongly agree”) to items regarding the likelihood of using the app frequently, the complexity of the app, ease of use, the need for support to use the app, whether people would learn to use the app quickly, confidence in using the app, and whether the user would have to “learn a lot” before using the app. The SUS has been applied to a wide range of technologies, has demonstrated good validity, can differentiate between usable and unusable systems, and it has demonstrated reliability even with small sample sizes [33-35].

Qualitative data on WorkingWell usability were obtained over time during technical assistance calls and the exit interview. Open-ended interview items included questions regarding whether users had difficulties logging in to the app; their confidence in using the app, given what they learned in the orientation session; whether it was easy or complicated to use the app and how they managed any challenges in app use; whether and which particular app components seemed confusing or not, along with recommendations for modifications; and what would have to happen for them to use the app regularly. In addition, in each technical assistance call, study participants were asked whether they were having any problems with the mobile phone per se and to describe them. This item was included so the research team could tease apart usability issues related to the phones rather the WorkingWell app.

#### Usage

Data on participant app usage were downloaded and monitored on a daily basis for quality assurance and app use tracking purposes. Data included participants’ daily number of navigations to the WorkingWell home screen and to the My Progress, Manage the Moment, Remind Me, and Rate My Day components of the app. To understand app usage in greater detail, open-ended interview items were developed and included in the technical assistance calls and the exit interview regarding when the participant tended to use the app and in what circumstances, and whether app use was integrated into a daily routine. Participants also were asked to describe a specific situation in which they used the app.

#### Usefulness

The usefulness of the WorkingWell app (ie, the ability to be used to achieve the user’s goal) was assessed in the poststudy survey by items regarding whether the app would help users remember why they want to work (motivation/job fit), manage better on the job (work self-efficacy), and connect with others who are supportive of their efforts to work (social support). Responses to these items were categorized as “agree,” “neutral,” or “disagree.” An additional item reflected whether the app would be useful in helping the user to stay on the job (rated on a 5-point Likert-type scale from 1=“strongly disagree” to 5=“strongly agree”). These items were developed to reflect potential mediators (eg, motivation, work self-efficacy, and social support) as well as anticipated outcomes (eg, job tenure), and the perceived usefulness of the app in addressing these variables. Qualitative data were obtained in technical assistance calls and exit interviews regarding app components or features that were most or least useful and the best and worst things about using WorkingWell, with prompts to provide detailed descriptions.

#### Feasibility

Feasibility of WorkingWell (ie, the likelihood that the app could be implemented successfully and used effectively) was determined by the percent of users completing the 8-week study. An exit interview item, developed for this study, solicited feedback on how likely the participant would be to continue using the app regularly (rated on a 10-point scale from 1=“not at all” to 10=“extremely likely”). Users were encouraged to provide recommendations for app modifications or additions. General impressions were obtained from users regarding the WorkingWell user support materials, technical assistance, and study procedures, with an eye toward framing future refinements to app support and the research protocol. Overall feasibility of the WorkingWell app will reflect consideration of findings regarding usability, usage and usefulness, along with study completion.

### Analysis

Quantitative data were entered into Qualtrics [36] databases and analyzed using SPSS version 24 [37]. Data were checked, cleaned, and managed by research staff, and item responses were recoded where necessary for consistent directionality. Descriptive statistics were computed for all items, and mean scale scores were calculated for the 6-item ease of phone use scale and the 7-item version of the SUS. Usage data were exported from the database within the app and compiled in spreadsheets for each participant. Usage plots for each participant, generated using SAS version 9.2 software [38], showed the number of navigations to various components of WorkingWell for each day within the 8-week study period.

Responses to open-ended interview items from technical assistance calls and exit interviews were exported for qualitative data analysis using the Dedoose software platform [39]. A framework approach was used to analyze qualitative data, given that the research team identified issues to investigate prior to study implementation (ie, usability, usage, usefulness, and feasibility) and developed interview items accordingly [40]. Therefore, some themes were identified in advance, while others were derived from the data as thematic coding progressed. Prior to the start of qualitative coding, the research team met to review the data, discuss the codes to be used, and informally code technical assistance calls in hard copies. Once a coding plan was established, two members of the research team coded text data, discussing and reconciling any disparate code identifications along the way. The research team prepared memos for themes reflecting study phone and app challenges, and for feedback on WorkingWell, user support materials and study procedures.

Trustworthiness of the qualitative analysis process and findings was established in multiple ways [41]. Five sets of randomly selected excerpts, coded by two members of the research team, were coded independently by the third member of the research team, achieving an average pooled kappa of .76 (range from .68 to .84), considered substantial agreement [42]. Differences were reconciled to achieve complete agreement in all cases. Due to the small sample size and exploratory nature of the study, all technical assistance calls and exit interview data were coded, rather than simply coding until data saturation was achieved. Trustworthiness was further established through peer debriefing and member checking [43] following contacts with study participants. Initial impressions, reviewed and discussed by the research team, were incorporated into subsequent interviews with later study enrollees, as well as into subsequent interviews with the same participant. Preliminary findings were reviewed in iterative cycles by independent stakeholders on the study’s Expert Advisory Group and actively working in the field.

### Ethics Approval and Consent

The study design and procedures were approved by the Dartmouth-Hitchcock Medical Center Committee for the Protection of Human Subjects (#00028834), the Massachusetts Department of Mental Health Central Office Research Review Committee (#2015-21), and the Vermont Agency of Human Services Institutional Review Board. Written informed consent was obtained from all participants at the beginning of the orientation session.

## Results

### Participant Characteristics

All participants (N=40) were included in the analysis of responses regarding background and demographic characteristics, and cellphone use. Participant characteristics are summarized in Table 1. More than half of the participants were male (24/40, 60%). The majority were age 55 or younger (28/40, 70%), white (32/40, 80%), had less than a 4-year college education (31/40, 78%), were employed part-time (39/40, 97%), had been working more than 6 months (24/40, 60%), lived independently (25/40, 63%), were never married (26/40, 65%) nor currently living with a partner (36/40, 90%), and were not parents (28/40, 70%). Of those reporting a known mental health diagnosis, 64% (16/25) indicated a diagnosis of bipolar or depressive disorder.

The vast majority of participants reported owning cellphones (38/40, 95%) and using them multiple times per day (33/40, 83%). They described using cellphones with ease (average rating of 3.78 on a 5-point scale). Cellphone data and ease of use are summarized in Table 2.

### Usability

Thirty-five of 40 enrolled participants completed 8 weeks of the study. Their average rating on the SUS scale was 3.93 (SD 0.77, range 1.57-5.00), as adapted for mobile phone apps, reflecting generally positive responses to usability items. Interestingly, the relationship between the ease of cellphone use (enrollment survey) and the SUS usability ratings was weak (*r*=.166).

**Table 1:** Demographic characteristics of study participants (N=40).

Participant characteristics		n (%)
**Gender**		
	Female	16 (40)
	Male	24 (60)
**Age**		
	18-35	11 (28)
	36-55	17 (42)
	≥56	12 (30)
**Race**		
	Caucasian	32 (80)
	African American	7 (18)
	Other	1 (3)
**Education**		
	High school diploma/General Education Diploma or less	18 (45)
	Vocational/military training/some college	13 (33)
	4-year college degree/graduate studies	9 (22)
**Current employment**		
	Full-time	1 (2)
	Part-time <30 hours a week	39 (98)
**Time at current job**		
	≤6 months	16 (40)
	>6 months	24 (60)
**Current living situation**		
	Own house or apartment	25 (63)
	House or apartment of parent, relative, or friend	9 (22)
	Residential treatment program or supervised living environment	6 (15)
**Ever married**		
	Yes	14 (35)
	No	26 (65)
**Currently living with spouse or partner**		
	Yes	4 (10)
	No	36 (90)
**Has children**		
	Yes	12 (30)
	No	28 (70)
**Mental health diagnosis^a^**		
	Bipolar disorder	11 (44)
	Schizoaffective disorder	5 (20)
	Depressive disorder	5 (20)
	Anxiety disorder/posttraumatic stress disorder	4 (16)

^a^n=25. Fifteen participants did not provide a specific mental health diagnosis.

**Table 2:** Participant-reported cellphone use at enrollment (N=40).

Cellphone use		Participants
**Cellphone access, n (%)**		
	Owns a cellphone	38 (95)
	No access to cellphone	2 (5)
**Cellphone type, n (%)**		
	Smartphone (phone with a data plan/internet)	28 (70)
	Basic mobile phone (phone with no internet)	10 (25)
	No access to cellphone	2 (5)
**Frequency of cellphone use, n (%)**		
	Multiple times per day	33 (83)
	One time per day or less	4 (10)
	No access to cellphone	3 (7)
**Ease of cellphone tasks,^a^ mean (SD)**		
	Typing	3.48 (1.36)
	Send a text message	3.93 (1.35)
	Access the internet	3.88 (1.45)
	Use an app	3.78 (1.42)
	Take pictures	3.98 (1.21)
	Use social networking sites	3.65 (1.44)

^a^Ease of cellphone tasks were rated on a 5-point Likert-type scale: 1 (“can’t do at all”) to 5 (“really well”), except for ease of typing, which was rated on a scale of 1 (“not at all easy”) to 5 (“extremely easy”).

Participants, in general, indicated that the WorkingWell app was “very easy,” “straightforward,” “simple,” and “user friendly” Some participants attributed this ease of use to the app navigation process and layout. One participant noted, “It was easy to pick up and learn, pretty straightforward. It was clear, laid out very well.” They described feeling as though there was no way to make a mistake in the app and if you did, it was easy to navigate away and attempt that task again. Participants who did not have extensive experience with mobile phones prior to participating in the study reported that they also found the app to be easy to use. For example: “I’m not a high-tech person. I don’t know anything about iPhones or how to download things, but the app is easy to use. It’s simple.”

When participants did report usability challenges, they tended to be framed as attributable to their personal challenges (eg, lack of familiarity with technology, confusion, forgetfulness) or to difficulties with the phone or app (ie, prototype layout or performance). One participant commented, “I am not very good with mechanical stuff, like setting passwords and stuff. To use the phone and app fully I have to get better at using computers and phones.” Overall, most participants were familiar with computers and/or mobile phones when they enrolled in the study. These participants seemed to have an ingrained sense of how to use basic phone functions and control settings, and to navigate to various components. The more experienced technology users often exhibited a more exploratory approach to familiarizing themselves with the phones (eg, navigating to all parts of the phone to see what was there), rather than the more regimented approach taken by many of those with less experience with this type of technology (eg, taking careful notes on navigation pathways).

Participants reported several types of app-related usability challenges regarding layout and content. One participant stated, “Navigating to the ‘My Tips’ section is kind of hard because there is so much there. And it’s hard to find the specific things I was looking for.” Another participant reported that large amounts of text were a challenge. One participant was unable to remember the meaning of some of the text, stating, “...I don’t remember what the skills mean. So, I don’t click on it [in Rate My Day] because I’m not sure if I used it.” Another participant described difficulty recognizing the implementation of specific skills in his own experience, stating, “Sometimes I don’t recognize what skill I used or didn’t use.”

One of the more common app-related challenges was prototype malfunction, for example, the appearance of unintended error messages sometimes combined with the app “freezing.” One participant described, “I was getting an error message and after that came up the screen wouldn’t do nothing. Only happened twice and then I turned it off and recharged it and it was fine.” These experiences seemed to sometimes be related to the use of the in-app “back” button. Many participants also described an app-related issue in which buttons were slow to respond or app screens were slow to load.

### Usage

Study participants were advised to use WorkingWell on the days they worked during the 8-week study period. Nearly all the participants worked part-time during the study. Twenty-eight were continuously employed, working at least 7 of the 8 weeks of their study participation. Two initiated job changes, and two were hospitalized during the 8-week study period and continued working, although fewer than the full 8 weeks. Three participants left their jobs but continued in the study while they looked for new positions. For the 31 participants reporting on the average number of hours worked per week at exit from the study, 65% (20/31) worked up to 20 hours per week on average and 35% (11/31) worked between 21 and 30 hours per week on average. These data reflect hours rather than actual days worked during the study, but they shed light on potential opportunities for app usage, given the instruction to participants to use the app on days when they were working.

Data on participant app usage varied considerably (Table 3). The most frequent navigations were to the WorkingWell home screen, which is the portal to using any of the app components. Next most used components were Rate My Day and My Progress, followed by Manage the Moment and Remind Me.

Several participants described a lack of time, energy, or focus as a personal barrier to using the app or completing app processes. As one participant described, “The big thing for me is...setting aside some time to actually work on it [the app]. I was going to do it at night, but I was too tired.” Other participants described difficulty remembering to use the app altogether. One individual described this experience as being related to symptoms of a possible medical condition, stating, “I think I have sleep apnea, so my memory is really bad and I’m always tired and I forget to do this.” Another participant described the interaction of infrequent app use with navigation difficulties saying, “I just can’t always remember, and it seems silly because there are only four [buttons]...I think if I worked more I would remember where everything is.”

### Usefulness

The WorkingWell app was described as useful by the majority of study participants (Table 4). Three-quarters of the participants (27/35, 77%) indicated that the app would help them remember why they want to work, 86% (30/35) agreed that the app would help them manage better on the job, and 57% (20/35) indicated that the app would help them connect with supportive others. The average rating of overall usefulness of the app was 3.74 (SD 0.92) on the 5-point Likert-type scale.

Participants described each component of WorkingWell as useful. In general, as one participant explained, “The best part was just knowing it [the app] was there. Knowing if I was in a funk, I had tools at my disposal.” Many appreciated the motivational quotes that appeared each time the app was opened, along with the affirmations that appeared in response to ratings in Rate My Day. For example, “I really like the inspirational quote. I would log in to the app just for the quote.” Participants appreciated the goal-setting feature of the app and the benefit of reflection at the end of the day prompted by completion of the Rate My Day component. A participate reported, “When you choose the three things to focus on for the week you narrow it down, so it is easier to focus on just a few things.” Participants indicated that using the app with their employment specialist to set up goals and review their progress would have been a good support.

Another participant described, “I like ‘Rate My Day’ because I can see how I’m doing and my progress.” These ratings are compiled into weekly progress reports, valued by a number of participants. “I’ve compared all my weeks in ‘My Progress’...It’s pretty cool that you can see patterns in your ratings.” Participants who integrated ratings of their days with ratings of coping skills began to see additional patterns emerge. “I find it [Rate My Day] more useful, so I am aware of what is going on...Now I think about why and about how to talk to other people about it, like my boss.” Participants were heartened by signs of progress: “‘My Progress’ is the most useful to me. It helps me be aware that I’m making progress and improving on tasks.” Some participants who did not find the goal-setting and rating features of the app useful were disappointed that they had to limit themselves to only three goals each week and were frustrated that they needed to change or re-enter their goals weekly.

Many participants described the Manage the Moment component of the app as useful. “I like the tips it gives you in detail and can help you apply these tips on the job.” Participants found the tips regarding interpersonal relationships helpful, for example, “How I used it to improve, like, talking to my boss instead of holding it inside.” Others found the tips in Manage the Moment effective in helping with managing symptoms on the job. For example, “The biggest thing for me is my anxiety and the tasks [tips] calm me down.” Other participants found the tips on lifestyle and wellness helpful, “I’ve been overtired, and you can’t work well when you’re like that and the app is reminding me how important it is that I get enough rest.” A few participants did not find this app component useful, indicating, “Some of the skills don’t really apply to my job.”

**Table 3:** Participant WorkingWell app usage during the 8-week study period (N=35).

Number of navigations to app component	Mean (SD)	Range
Home screen	72.0 (43.5)	8-178
My Progress	37.8 (25.2)	1-98
Manage the Moment	16.9 (16.2)	1-55
Remind Me	14.3 (12.7)	1-42
Rate My Day	41.2 (31.0)	1-107

**Table 4:** WorkingWell app usefulness and feasibility ratings at study endpoint (N=35).

Usefulness and feasibility		Participants	
**The WorkingWell app would help me remember why I want to work, n (%)**			
	Agree		27 (77)
	Neutral		7 (20)
	Disagree		1 (3)
**The WorkingWell app would help me manage better on the job, n (%)**			
	Agree		30 (86)
	Neutral		5 (14)
**The WorkingWell app would help me to connect with people who are supportive of my efforts to work, n (%)**			
	Agree		20 (57)
	Neutral		14 (40)
	Disagree		1 (3)
Overall, how useful would this app be in helping you stay on the job?^a^, mean (SD)		3.74 (0.92)	
How likely would you be to continue using this app regularly?^b^, mean (SD)		7.99 (1.89)	

^a^Usefulness of the app in helping to stay on the job rated on a 5-point Likert-type scale: 1 (“strongly disagree”) to 5 (“strongly agree”).

^b^Feasibility (ie, the likelihood) of continuing to use the app regularly rated on a 10-point Likert-type scale: 1 (“not at all”) to 10 (“extremely likely”).

Many participants reported that the Remind Me feature was useful. For example, “I like to use the text message reminders to send positive messages to myself. It gives you more positivity during the day.” Several indicated they used the Remind Me component to break their job tasks into small steps to make tasks more manageable. Those participants who did not find text message reminders helpful tended to already use other phone features to set reminders, such as the calendar or alarm feature. One participant suggested that one bad thing about reminders is that, “People can get dependent on them.”

### Feasibility

Thirty-five of the 40 original participants (88%) completed the 8-week study. The ratings of the 35 study completers regarding the likelihood of using the app regularly were quite positive, with a mean of 7.99 (SD 1.89) on the 10-point scale. The five participants who did not complete 8 weeks of the study participated for a mean of 4.8 weeks (range 2.5-7 weeks) and ended participation for various reasons, including changes in work schedule that precluded participation.

Some participants mentioned that the user support materials provided to them at orientation by the research staff (eg, WorkingWell User Guide, study phone user guide) were helpful supplementary materials to the in-person orientation sessions. They served as references to consult if participants forgot how to perform a specific function in the app. Other participants described using support materials beyond those created by the research staff, such as creating individualized step-by-step instructions on how to navigate the app or checking out a phone user manual from the library to learn more about the study phone. Several participants discussed issues they had with the user support materials. One participant noted, “Papers and papers about something electronic just make me nervous.”

Some participants reported seeking technical assistance from agency staff such as their job coach or employment specialist or from other support persons such as rehabilitation coordinators or counselors. They indicated that having this resource was important, as “hands-on instruction is best.” Participants also cited receiving assistance from family and friends. This help seemed to focus primarily on difficulty performing tasks on the study phones rather than with the WorkingWell app. Several participants noted that they reached out to other participants in the study to either request technical assistance or to provide it. Study participants suggested several changes to study procedures that would improve their use of the app. Some participants mentioned that having the app on their own phone, rather than a separate study phone, would make it easier to remember to use the app daily and incorporate it into their routine more conveniently.

## Discussion

### Principal Results

In this study we posed four questions regarding the usability, usage, usefulness, and feasibility of the WorkingWell mobile app for individuals with mental illness coping on the job. Our findings, largely positive, support the potential use and benefit of an app such as WorkingWell for this target population. Data from study participants suggest modifications that will improve the app and that are relevant to study design and procedures for next-step efficacy testing. Recommendations for modifications are provided as they reflect findings discussed subsequently.

The weak relationship between ratings of ease of cellphone use and usability of the WorkingWell app (SUS) is a positive finding. It suggests that participants’ experiences of using WorkingWell is independent of their ability to use a cellphone. Both novice and experienced cellphone users were equally able to use the app. WorkingWell is designed such that cellphone familiarity is not essential to use of the app.

Although WorkingWell received generally high marks and positive feedback on usability, some users found it to be too “wordy.” Some were unsure of the meaning of some of the coping skills and consequently had difficulty applying these tips in their daily lives. These findings are consistent with recommendations for critical design elements in previous research with individuals with mental illnesses: include a singular focus, simple architecture, prominent contents, explicit navigation, and inclusive hyperlinks [28]. Researchers have suggested the value of testing language used with potential end users, in this case, individuals with mental illness, who may have idiosyncratic notions regarding the meaning of commonly used words and phrases [28]. These findings suggest the potential benefit of reducing the volume of text in the next iteration of WorkingWell and conducting more extensive usability testing regarding the language used. In addition, there were several reports of app malfunction. Modifications were made to WorkingWell during the study as problems were identified.

Usage varied considerably among WorkingWell participants, ranging from minimal usage to, most likely, several times per work day. Subsequent visual inspection of graphs of individual navigations to the home screen over the course of study involvement suggested several diverse patterns ranging from those whose use peaked at the beginning of the study and then dropped off to those whose use was fairly consistent over time. During qualitative interviews some users reported that they forgot to use the app or were too tired at the end of the day. Therefore, it may be helpful for the user to set personalized text message reminders as a routine to encourage use of the app at a time that is convenient. The benefits of personalized contacts in promoting higher response rates to Web- or internet-based surveys, for example, have been demonstrated [44]. If the user is accessing WorkingWell in collaboration with a supervisor, colleague, or employment specialist to improve on-the-job functioning, they might set reminders together. It may also be helpful to program in routine reminders for app use that could be personalized by the user and modified over time as schedules and routines change.

Users provided positive feedback on the usefulness of the app. They valued the motivational quotes and supportive feedback on daily ratings. Study participants enjoyed setting goals, monitoring progress and reflecting on patterns over time in challenges, coping efforts, and ratings. Fewer found the app helpful in connecting with supportive others, suggesting the potential benefits of modifications to facilitate data sharing and the solicitation of feedback from others to increase interactivity. Some users suggested that setting goals and monitoring progress with employment specialists, for example, would be useful. Study participants also recommended building in greater capacity to tailor the app to make it more relevant to specific job sites and responsibilities.

Given the finding that 35 of 40 study participants completed the study, and including consideration of findings regarding usability, usage, and usefulness, WorkingWell was found to be a feasible approach to helping individuals with mental illness to cope on the job. The findings that the majority were continuously employed throughout the study and that those who were not had plans to seek new positions while using the app suggest that WorkingWell may be efficacious in sustaining both motivation to work and employment. The WorkingWell app was found to be useful by participants who had only been employed for a short time as well as those who had been employed for a longer period. This suggests the app can be useful not only for those coping with the stress of a new job, but for those navigating the challenges of sustaining employment over time. Although a job may become less challenging over time, as a person learns and masters the day-to-day expectations and routines, the impairments conveyed by serious mental illnesses may not change over time (eg, memory or attention deficits). Consequently, the WorkingWell app may be useful throughout an individual’s employment.

### Limitations

The developmental mixed-methods approach of this study allowed us to look closely at the usability, usage, usefulness, and feasibility of WorkingWell. Future research on efficacy and effectiveness will require larger, more diverse samples, with a randomized controlled trial design, a longer follow-up period, and the use of targeted standardized outcomes [45]. A larger sample size would allow us to stratify the sample by individual characteristics that may be associated with outcomes to increase statistical power. Assessing the impact of the WorkingWell app in real-world practice would require participants using their own phones and data plans, as well as the testing of various levels of orientation and support to the app. Facilitating use of the app on participants’ mobile phones, rather than study-provided phones, may promote increased and routine use of the app. Alternatively, participants may find their phones or data plans burdened by WorkingWell app use. Further research will also enable us to explore the use and effectiveness of the app in diverse employment contexts. The WorkingWell app will require modifications and additional usability testing to address the recommendations provided by participants in this study.

### Comparison With Prior Work

Prior research has provided evidence of the penetration and use of mobile phones and mobile technology by individuals with mental illness [46]. In this study, participants—admittedly willing volunteers—seemed interested in using WorkingWell and generally put it to use. The participants appeared comparable to those in other studies of supported employment [47], suggesting the potential generalizability of our findings to this population. Moreover, our findings suggest the potential usability and usefulness of WorkingWell for the larger population of individuals receiving supported employment services (eg, people with autism, first-episode psychosis, or cognitive deficits). The core elements of WorkingWell (eg, staying motivated, goal-setting and progress monitoring, managing stress, remembering job tasks and responsibilities, and getting along with others) reflect challenges for many employees in many workplaces, suggesting the potential usefulness of the app for workers and supervisors across settings. The sound conceptual and theoretical underpinnings of WorkingWell enhance the likelihood of its effectiveness and broad applicability [48].

The process by which we developed the WorkingWell app was consistent with recent recommendations of experts in the field for the development of mHealth interventions for individuals with serious mental illness [49]. WorkingWell was developed to meet an unmet need for follow-along supports in the workplace for individuals coping with mental illness on the job. Stakeholders, including individuals with mental illness, experts in supported employment and technology-based interventions, and experienced designers were involved in every step of the development and testing of WorkingWell [30,31]. Because of their contributions, we are well-positioned to transition to future effectiveness studies once user-recommended modifications have been made.

Our findings are consistent with those of other studies of emerging mHealth and eHealth interventions targeting individuals with serious mental illnesses in terms of feasibility and acceptability [45]. Measures of feasibility and acceptability described in a recent meta-analysis of previous studies of similar technology-based approaches with diverse populations of individuals with mental illnesses include frequency of intervention use over time, response rates, attrition rates, study retention, proportion of devices that were returned undamaged, participant-reported usability, and responses obtained in qualitative interviews soliciting participant feedback [45]. Overall feasibility in our study is measured by overall study completion rate (35/40, 88%), positive scores on the item pertaining to the likelihood of continued use of the app, and feedback obtained via quantitative measures and open-ended interview items regarding usability, usage, and usefulness of WorkingWell comparable to the approaches to feasibility and acceptability used in prior studies. Our study completion rate of 88% (35/40) is in keeping with findings of participation, adherence, and completion in other similar studies that ranged from 70% to nearly 90% [45]. Differences across studies seem to be related to the targeted behaviors or symptoms, the level of support (eg, use of the app in addition to peer support or in-person sessions), and study characteristics such as target population and issues of research measures and methods [45].

### Conclusions

The WorkingWell mobile app is a feasible approach to providing accessible, as-needed employment support for individuals with mental illnesses as they cope with the expectations, tasks, and social demands of work. Although WorkingWell was developed with extensive input from research, training, and practice experts, along with input from and usability testing with individuals with mental illnesses, the app would benefit from additional modifications to address recommendations from our in-depth testing. Further controlled research with larger samples, more diverse in individual characteristics (eg, work history and illness severity), and workplace settings (eg, more or less structured, routinized positions) is essential to demonstrating the effectiveness of the app in enhancing employment tenure and job satisfaction. Study protocols that include assessment of potential moderating factors, such as prior work history and illness severity, and mediating factors, such as work self-efficacy and job satisfaction, will contribute to our understanding of the ways in which supportive, technology-based tools like WorkingWell contribute to positive outcomes such as job tenure.

## Abbreviations

IPS: Individual Placement and Support
SUS: System Usability Scale
